# Performance of wild animals with “broken” traits: Movement patterns in nature of moose with leg injuries

**DOI:** 10.1002/ece3.9127

**Published:** 2022-07-31

**Authors:** Andrew P. Hendry, Cedar A. Hendry, Aspen S. Hendry, Heather L. Roffey, Michael A. Hendry

**Affiliations:** ^1^ Redpath Museum and Department of Biology McGill University Montreal Québec Canada; ^2^ Private Citizen; ^3^ Napa California USA

**Keywords:** functional traits, game camera, lameness, natural selection, trailcam, ungulate

## Abstract

Organismal traits are presumed to be well suited for performance in the tasks required for survival, growth, and reproduction. Major injuries to such traits should therefore compromise performance and prevent success in the natural world; yet some injured animals can survive for long periods of time and contribute to future generations. We here examine 3 years of camera trap observations along a remote trail through old‐growth forest in northern British Columbia, Canada. The most common observations were of moose (2966), wolves (476), and brown bears (224). The moose overwhelmingly moved in one direction along the trail in the late fall and early winter and in the other direction in the spring. This movement was clustered/contagious, with days on which many moose traveled often being interspersed with days on which few moose traveled. On the video recordings, we identified 12 injured moose, representing 1.4% of all moose observations. Seven injuries were to the carpus, three were to the antebrachium, and two were to the tarsus—and they are hypothesized to reflect damage to ligaments, tendons, and perhaps bones. The injured moose were limping in all cases, sometimes severely; and yet they did not differ noticeably from uninjured moose in the direction, date, contagiousness, or speed of movement along the trail. We discuss the potential relevance of these findings for the action of natural selection in the evolution of organismal traits important for performance.

## Graphical abstract

Major injuries should compromise organismal performance and prevent success in the natural world. Using camera trap video recordings along a remote trail in northern British Columbia (Canada), we identified 12 moose with leg injuries, including 7 to the carpus, 3 to the antebrachium, and 2 to the tarsus. The injured moose were limping in all cases, sometimes severely. However, we here show that these moose did not differ noticeably from uninjured moose in the direction, date, contagiousness, or speed of movement along the trail. We discuss implications for the efficacy of natural selection.

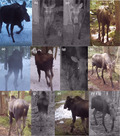

## 1 INTRODUCTION

Organismal traits have evolved over long periods of evolutionary history to improve performance in tasks (e.g., predator avoidance, food acquisition, long‐distance migration) that increase evolutionary fitness—that is, the combination of survival and reproductive success (Arnold, 1983; Cain, 1964; Darwin, 1859; Hendry, 2017). As such, dramatic deviations of individual trait values from the typical distribution of trait values are expected to decrease fitness, at least in most cases. Yet it has proven difficult to assess the fitness costs of such “outlier phenotypes” because—following the underlying logic—they should compromise survival to such an extent that the individuals that bear them should be only rarely observed (Haller & Hendry, 2014). Hence, assessments of how trait values might be optimized for performance in a given environment typically involve experimental manipulations, such as translocations to different environments (Hereford, 2009; Leimu & Fischer, 2008), hybridization experiments (Rieseberg et al., 1999), induced mutations (Ahloowalia & Maluszynsk, 2001), or direct phenotypic manipulations (Sinervo et al., 1992). The artificial state of these approaches leaves uncertain the fitness costs of outlier phenotypes under more natural conditions.

Several situations arise, however, where outlier or “exceptional” phenotypes are produced more naturally, and thus can be assessed for their effects on performance, survival, reproductive success, or fitness. One situation occurs when individuals with phenotypes that evolved in one environment naturally disperse to a different type of environment. Studies examining such out‐of‐place individuals often document reduced survival or reproductive success (Nosil et al., 2005), although it can be unclear whether their problems result from mismatched traits or from some more general costs of dispersal or establishment. Another situation occurs when hybridization generates trait values atypical for either group; where, again, fitness costs are frequently observed (Rundle, 2002). Although both of these situations generally reveal fitness costs of outlier phenotypes, some exceptions are known, such as when extreme display traits are favored by sensory bias (Ryan & Keddyhector, 1992), when extreme “transgressive” phenotypes happen to match new fitness peaks (Rieseberg et al., 1999), or when environments are rapidly changing (Bell & Gonzalez, 2009).

In the above situations, outlier trait values typically represent extremes along normal axes of variation—that is, the traits are not “broken.” Broken traits, by contrast, get at the heart of what is important about a trait itself, and are therefore particularly interesting to examine. One relevant situation occurs for organisms that show limb autotomy. For example, some lizards, salamanders, and scorpions can drop their tails when attacked by predators, many decapod crustaceans (e.g., crayfish) can do the same for their claws, and some sea cucumbers can eject parts of their gut to distract predators (Maginnis, 2006). These cases of broken (in fact, missing) traits do reveal fitness consequences, such as reduced growth or survival; yet, evolution has presumably led to compensatory structures or behaviors that minimize the costs incurred when the trait is lost. The situation is presumably quite different for unplanned injuries.

Trait changes resulting from injuries can take many forms. Perhaps most frequently, animals can have cuts, tears, or gashes in their soft tissues—which often heal. Other more “structural” injuries influence ligaments, tendons, joints, or hard structures such as beaks, teeth, or bones. Injuries to some of these hard structures are well documented as they leave lasting signatures evident even long after death. Studies of structural injuries include birds that break their beaks (Slevin et al., 2016) and carnivores that break their teeth (Van Valkenburgh & White, 2021). Further, animals can break any number of bones (Stephens et al., 2018; Woodman, 2013), such as by falling from trees or cliffs or via damage from predators or humans (e.g., bullets, cars, traps, snares) or human structures (e.g., cattle guards). Many such injuries are fatal in the short term (e.g., via blood loss) or in the moderate term (e.g., via starvation or increased predation risk)—and yet some organisms live far beyond their initial injuries, which then heal to varying degrees. For instance, healed bones have been reported in many animals, including dinosaurs (Hao et al., 2020), primates (Bulstrode et al., 1986; Lovell, 1991), small mammals (Stephens et al., 2018), and—of particular relevance to the present study—artiodactyls like deer (Grandstaff et al., 2015).

What is the impact of such (partially) healed injuries on the performance of individual organisms (Lovell, 1991)? In humans, the outcomes are highly variable—with some athletes completely recovering performance despite horrific broken limbs, and other athletes never again performing at the same level even from only modest damage (Frangiamore et al., 2018; Johns et al., 2021). In domestic animals, many individuals can remain ambulatory despite missing limbs (e.g., three‐legged dogs), whereas others are often euthanized after even minor lower limb breaks (e.g., horses). Humans and domestic animals are typically afforded advanced health care where they can recover and remain in sheltered environments away from predators and with ample food. What of wild organisms not receiving the benefits of such “soft landings” after structural limb injuries?

**FIGURE 1:**
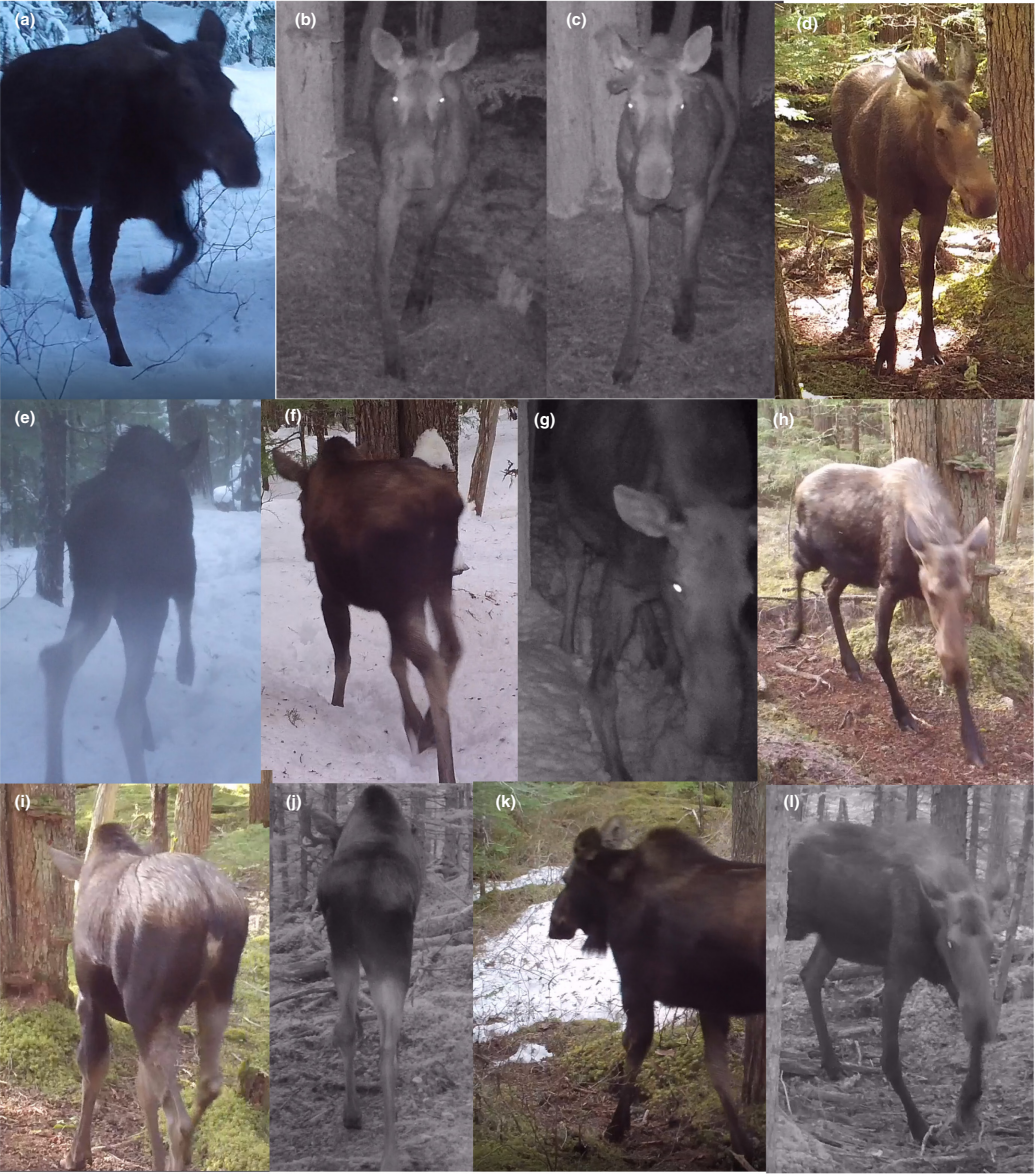
Cropped screen captures from camera trap videos of the 12 moose with injuries. Hypotheses about the nature of these injuries are listed in Table 1.

Our study was motivated by observations of moose with leg injuries recorded on camera traps in a remote area of northwestern British Columbia, Canada. These injuries were typically evident as enlarged joints or limbs, and/or striking angular limb deviations (i.e., medial or lateral). Most of the injuries caused the moose to limp moderately to severely (at least to our subjective interpretation), and yet we observed the same injured moose passing multiple camera traps, and at least some of those moose appear to have been present in multiple years. The current study takes advantage of a recent (2017–2020) deployment of high‐quality camera traps along a well‐defined trail passing through old‐growth forest to examine the movement patterns of injured moose under natural conditions. Observations of 12 injured moose (Figure 1) provided an opportunity to consider the movement behavior of injured moose relative to uninjured moose.

## 2 DATA COLLECTION

We used 10 camera traps to instrument a well‐defined animal trail in the Kispiox Valley of northwestern British Columbia, Canada. We do not provide a precise location for the study so as to protect sensitive wildlife; fortunately, precise locations are not relevant to the questions we address. The part of the trail we studied is 3.3‐km long and runs midway between a lake and a river, which are 600–800 m from each other for most of that distance. The wide ridge along which the trial passes is dominated by coniferous forest, mostly spruce, hemlock, and fir; and it appears to offer few foraging opportunities for moose or deer. Instead, the trail connects two wide river floodplains that are dominated by alder, willow, and popular; areas that do provide foraging opportunities. Thus, our impression from personal observations before camera trap placement was that the trail was mainly used for transit between the upstream and downstream foraging areas. Our study focused on how animals moved along this trail during those transit events.

Our goal was to quantify various aspects of animal behavior and movement, and so we set the camera traps to record videos (see Caravaggi et al., 2017) rather than still images. The camera traps were Reconyx UltraFire XR6, which record videos at 1080P using two cameras—one specialized for color recordings during the day and the other specialized for monochrome recordings at night. Night recordings are illuminated with built‐in “covert” “no‐glow” infrared lights expected to be invisible to mammals (Trolliet et al., 2014). The lack of moving parts means that the cameras are asserted (by the manufacturer) to be silent, further minimizing disturbance to animals. (As an aside, animals—especially wolves—occasionally “startled” when a camera turned on, and so the cameras might not be entirely covert).

**FIGURE 2:**
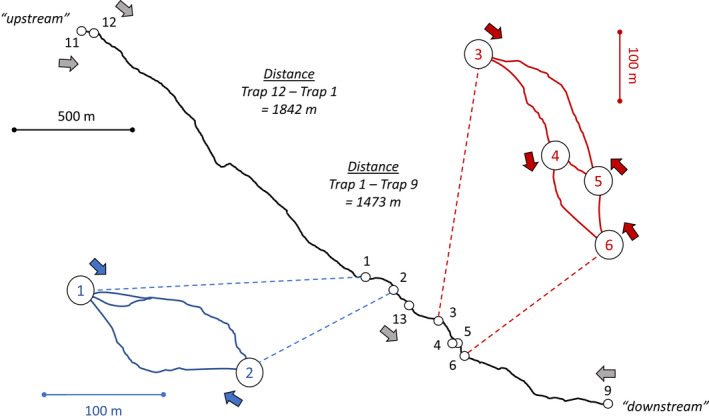
The course of the trail (solid lines) and the camera trap locations (open circles) as mapped by GPS—actual coordinates are not provided in order to protect sensitive wildlife. Numbers in the circles correspond to the camera trap numbers as reported in this paper. Colored (blue and red) insets show magnified portions of the trail with dense placement of camera traps. Filled arrows beside each camera trap number show the direction the camera is pointed. The two ends of the trail are referred to as “upstream” and “downstream” throughout the text, a reference to the river (not shown) that runs roughly parallel to the trail.

A first set (“set 1”) of six camera traps (numbered 1–6) was deployed on August 8, 2017. These traps were placed facing obvious splits in the trail in order to record the side of the split taken by each animal (side‐biases at these trail splits will be the focus of a subsequent paper). When referring to the different “sides” of each split, we use “left” and “right” in reference to the downstream direction of flow in the adjacent river. Two different groups of trail splits were instrumented with the cameras (Figure 2). In the upstream group, Trap 1 pointed downstream at a three‐way split, with the middle path rejoining the left path after 25 m. The right path then rejoined the middle/left path after 150 m. Trap 2 was pointing upstream to record this rejoining of the right and middle/left paths. The downstream group of trail splits was more complex, with four splits over approximately 300 m—each instrumented with one camera (Figure 2). This first set of six camera traps was serviced (batteries checked, videos downloaded, memory cards replaced) on August 7, 2018. They were again serviced on July 15–18, 2019, and on August 8, 2020.

A second set (“set 2”) of four camera traps (numbered 9, 11, 12, 13—missing numbers correspond to traps used for other purposes) was first deployed on August 10, 2018. Trap 12 was placed facing a short steep hill where the trail descended to the upstream river flats. The other three traps were placed to record both the trail and a bear‐rubbing tree immediately beside the trail. Trap 11 was placed at the upstream end of the trail, 72 m upstream of Trap 12. Trap 9 was placed at the downstream end of the trail, 470 m after it descended to the downstream river flats. Trap 13 was placed between the two groups of splits that had been instrumented in the first set of traps (Figure 2). This second set of traps was serviced on July 15–18, 2019, and on August 8, 2020.

**FIGURE 3:**
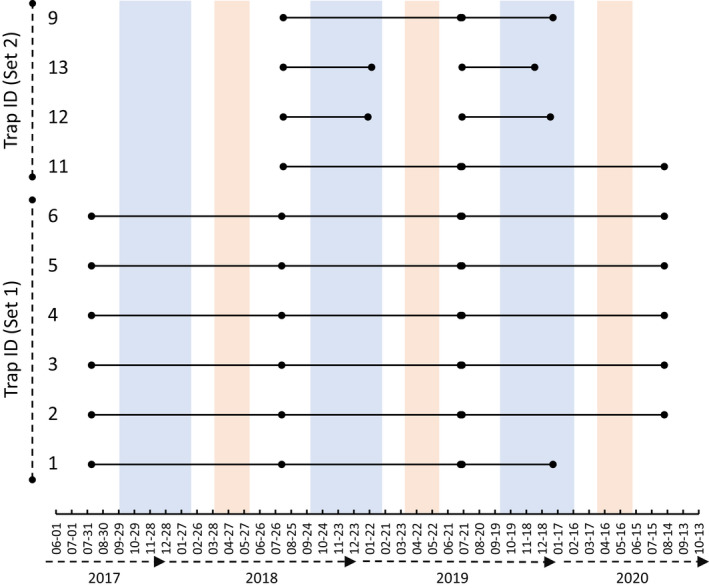
Dates of camera trap placement (earliest black dot for each camera) and servicing (subsequent black dots) for each of the two sets of camera traps. Camera traps 12 and 13 stopped recording before servicing each year, as indicated by black dots that are not immediately followed by a black line. Camera traps 1 and 9 stopped recording before servicing in the final year, as indicated in the same way. Blue and orange bars indicate periods designed in the text as the primary “fall” (October through January) and “spring” (April and May) movement periods of moose on the trail (as mirrored with similar bar colors in Figure 5).

To the extent possible, all camera traps were deployed with the same settings: triggered by animal movement (default = “high” sensitivity), maximum video length of 30 s, and a delay between consecutive recordings of 5 s. However, three deviations from standard settings arose owing to battery differences, apparent programming differences between some cameras, and adjustments to problems encountered. (1) Over the first year (fall 2017–fall 2018), the cameras would turn off the video whenever an animal stopped moving during the 30 s recording interval. In subsequent years, all cameras were programmed to record for 30 continuous seconds regardless of whether or not the animal continued moving. (2) The second set of cameras insisted on taking a single picture before each video recording, and we were unable to remove this setting. Hence, both the initial photo and the subsequent video from these cameras needed to be examined to identify the animals. (3) The first set of cameras was equipped with Energizer Ultimate Lithium AA‐cell batteries, which worked continuously for the entire time period (except that camera trap 1 ran out of batteries on January 8, 2020). In hopes of improving sustainability, the second set of cameras was equipped with Tenergy NiMH rechargeable AA‐cell batteries, which were often drained before the spring (Figure 3).

When viewing the videos, the following baseline information was recorded for each individual animal: date, time, direction of movement (upstream, downstream, or neither), and the species of animal (the data are published here: https://doi.org/10.5061/dryad.xd2547dkk). Additional information recorded will be discussed below in the context of research questions we sought to answer. Several potential limitations were revealed during this video screening process. First, some recordings did not capture any animal—seemingly because the animal passed by too quickly between when the camera was triggered and when it started recording (about 1 s). This fact was usually evident based on branches moving in the video, or fresh tracks in the snow relative to the previous recording. Second, the videos were foggy in some instances, which precluded the accurate collection of some information. Third, martens and fishers were difficult to distinguish as they moved rapidly through the videos and so are grouped for analysis.

After excluding all recordings that took place while deploying and servicing the cameras, we obtained 4341 total discrete observations: that is, every animal observed on every recording. We next excluded 241 observations (5.6% of 4341) of our friends and family, and also 25 observations (0.6% of 4341) of other humans. The observations of other humans were all from Trap 9, which is located at downstream end of the trail and is therefore more accessible. In short, beyond a few recordings at the first trap on the trail, no humans other than ourselves were observed over the entire 3 year period. Of the remaining 4075 observations, we next excluded 128 (3.1% of 4075) “immediate repeat” observations: that is, instances where sequential recordings from a single camera trap were clearly of the same individual animal. We could not exclude potential “later repeats,” which could include the same individual animal recorded on different camera traps, or the same individual recorded hours later on the same camera trap. Of the remaining 3947 observations, we next excluded nine observations (0.2% of 3947) of domestic dogs, which were always with humans, as well as 164 recordings (4.2% of 3947) where no animal could be seen. In nearly all of these latter cases, moving branches indicated that an animal had passed too quickly to be recorded. Finally, we removed all observations of birds because they were too few to be worth analyzing: three observations of grouse and one of a Stellar's Jay. The remaining 3770 observations were used for subsequent analyses.

Our camera trap deployments were optimized for studying animal movement along a single trail and, hence, several other types of inferences would not appropriate. First, we do not estimate population sizes because doing so requires other designs for camera placement (Palencia et al., 2021), and because our different traps often recorded the same individual animals (only some of which could be identified as such). Second, the animals often traveled together or in temporal clusters (details below), which meant that they do not represent independent observations. This non‐independence rules out most inferential statistics, such as confidence intervals or p‐values; and instead invites more descriptive statistics, such as counts and percentages. Importantly, then, our inferences are restricted to the set of individuals that we recorded—and should not be generalized to some larger “population” of individuals moving along this trail in other years or along other trails.

## 3 BASIC PATTERNS

**FIGURE 4:**
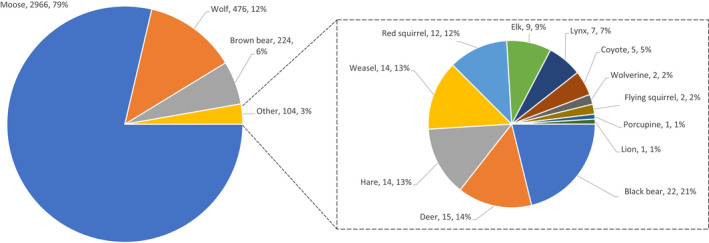
Numbers and percentages of the total observations of non‐human animals on the trail. These totals are based on all camera traps across all 3 years.

We first considered the species of animal that were commonly using the trail by tallying all observations of non‐human animals across all cameras in all years. These tallies include an unknown number of repeat observations of the same individual animals across space (different traps) and time (different seasons and years). Hence, the tallies reflect general use of the trail, rather than any sort of population size estimate. These tallies revealed that moose were the primary users of the trail (78.7% of all observations), followed by wolves (12.6%) and brown bears (5.9%), and then by lower numbers of many other species (Figure 4). Several additional comparisons help to highlight aspects of fauna using the trail. First, we had an order of magnitude more recordings of brown bears (224) than black bears (22). Second, we had nearly two orders of magnitude more recordings of wolves (476) than coyotes (5). Third, all observations of elk were from June of 2018. Fourth, very few deer were observed on the trail and only one mountain lion was recorded.

Qualitative observations from lower‐quality camera traps deployed in previous years suggested that moose were using the trail for continuous (i.e., without stopping) transit along the ridge connecting the upstream and downstream river flats. Furthermore, those transits appeared to be primarily downstream in the late fall and early winter but primarily upstream in the spring. To here quantify those patterns, we examined data from the five camera traps (traps 2–6 in set 1) that had continuous recordings for the entire three‐year period (Figure 3). Camera traps from set 2 were excluded because they were not deployed in the first year, and Trap 1 was excluded because it stopped recording in January of the third year. For each moose recorded on traps 2–6, we documented whether it was moving downstream (961 observations) or upstream (792 observations), or whether the direction of movement was unclear (7 observations). The latter situation was typically invoked when moose were moving perpendicular to the trail.

**FIGURE 5:**
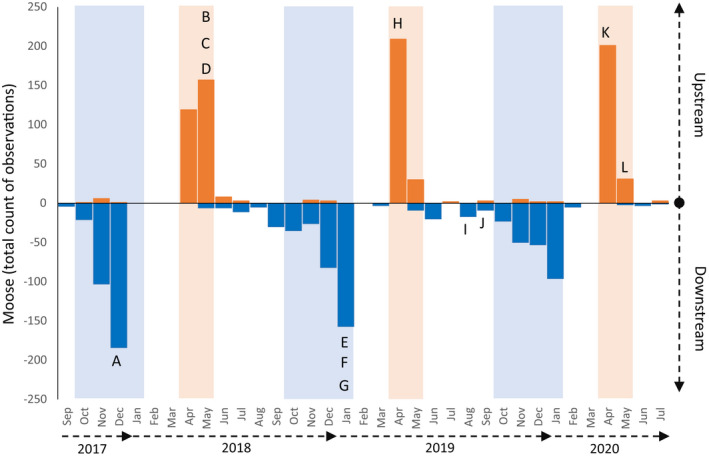
Seasonal movement patterns of moose based on data from the camera traps that had complete recordings across all 3 years (i.e., traps 2–6). Moose were nearly always moving in the downstream direction (dark blue bars) in the fall period (light blue range) and in the upstream direction (dark orange bars) in the spring period (light orange range). Capital letters indicate the month of observation and the direction of movement of the 12 injured moose shown in Figure 1 and Table 1.

These new quantitative data confirmed our earlier qualitative observations. That is, moose typically transit the trail without lengthy pauses (see estimated rates of transit below) and that—overwhelming—they moved downstream in the late fall and early winter but then upstream in the spring (Figure 5). Furthermore, almost no moose were observed on the trail in the late winter (February and March), and only a few moose were observed in the summer (June and July). Beyond this exceptionally strong pattern of seasonal movement, a few interesting nuances are worth noting. First, downstream movement ceased much earlier in 2017 (December 19 was the last observation) than in subsequent years. For instance, 157 moose moved downstream in January 2019 (the last observation was January 26) and 96 moved downstream in January 2020 (the last observation was January 30). Second, upstream movement was distributed later in the spring of 2018 than in the spring of 2019 and 2020 (Figure 5).

**FIGURE 6:**
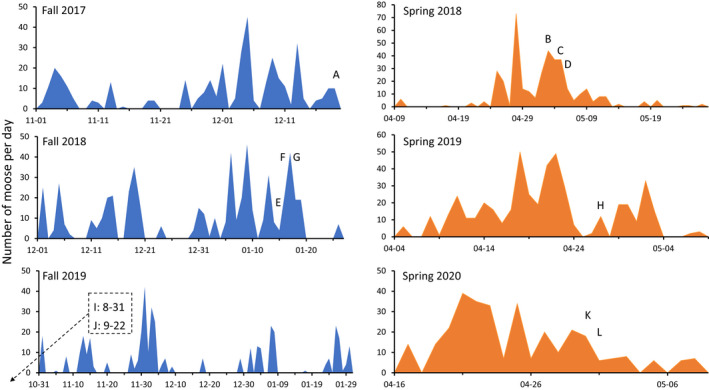
Daily movement of moose based on data from all camera traps. The *x*‐axis differs among plots according to the time range of movement. The data labels are every 10 days in all panels to enable quick comparison of the time intervals. Capital letters indicate the date of observation of the 12 injured moose shown in Figure 1 and Table 1. Note that injured moose I and J moved downstream earlier in fall 2019 than the period illustrated in the panel.

Another striking pattern was that—within a season—the movement of moose was strongly clustered or “contagious” (Figure 6). That is, some days with many moose observations (up to 73 in a single calendar day) were often interspersed by several days with few or no moose observations. In fall 2017, for example, only five observations were made on December 2–3 and only four observations were made on December 6–7; yet 73 observations were made on the two intervening days. The degree of this contagiousness appeared to vary across seasons and years. For example, compared with upstream movement in the spring, downstream movement in the fall was more spread out into numerous smaller clusters separated by longer periods (Figure 6). Also, movement in fall 2019 was much more spread out in this regard than was movement in fall 2017 and fall 2018 (Figure 6). Again, these qualitative descriptions are made without the accompanyment of inferential statistics.

## 4 HOW DO INJURED MOOSE COMPARE?

We observed some instances of cuts or sores on moose, but we here focus on obvious structural leg injuries that are readily identifiable across video recordings on multiple camera traps. We first screened all recordings on all camera traps to note such injuries. We then carefully re‐screened all other videos within 12 h of a given observation of an injured moose. This re‐screening was helpful because an injury sometimes was not immediately noticed during initial screening, and yet could be confirmed via slow motion and magnification of the videos. In all cases, we only retained videos of injured moose where the injury was visible, and where we were confident that it was the same individual moose with the same injury within a given season.

This screening procedure identified 12 moose with obvious leg injuries (Figure 1; Table 1). The videos are supplementary material video S1 and are also posted at www.youtube.com/watch?v=ly1jVA6w5WM. Seven of the injuries were to the right carpus, which is homologous to the human wrist, and these were characterized by lateral displacement and possible hyperextension (6 injuries) or medial displacement (1 injury). Three of the injuries were to the antebrachium, which is homologous to the human lower arm, and were characterized by swelling and possible bone remodeling. The remaining two injuries were to the left hock (tarsus), which is homologous to the human ankle, and were characterized by enlargement near the Achilles tendon attachment. These 12 injured moose were distributed across all years and both seasons (fall and spring), and the total number of confirmed observations (33) plus the total number of additional likely observations (8) together represented 1.4% of the total number of moose observations (2966).

Based on the sex, size, and appearance of the moose, as well as the location and nature of the injuries, we are confident that the 12 injured moose include at least nine different individuals (Table 1). That is, based solely on examination of the videos, we cannot exclude the possibility that injured moose B (moving upstream in spring 2018), injured moose H (moving upstream in spring 2019), and injured moose L (moving upstream in spring 2020) are the same individual. Similarly, we cannot exclude the possibility that injured moose I (moving downstream in fall 2019) and injured moose K (moving upstream in spring 2020) are the same individual. It would be exciting to thereby infer inter‐annual survival of injured moose. However, we also cannot be c onfident that those moose are—in fact—the same individual. Hence, we proceed by treating each of the 12 injured moose separately, while noting that they might represent as few as nine individuals.

**TABLE 1:** Summary information for the 12 injured moose.

Moose	Date	Direction	Traps observed	Injury location and hypothesis
A	December 19, 2017	Down	1,2,3,4,6	Right carpus—hyperextension and lateral displacement. Possible ligament damage
B^a^	May 13, 2018	Up	6,4,3,2,1	Right carpus—hyperextension and lateral displacement. Possible ligament damage
C	May 05, 2018	Up	2,1	Right carpus—medial displacement. Possible medial collateral ligament damage
D	May 06, 2018	Up	6,4,3,2,1	Right carpus—swelling, stays in flexed position. Possible bone remodeling
E	January 15, 2019	Down	1,2,13,3	Left tarsus—posterior swelling. Possible Achilles tendon damage
F	January 16, 2019	Down	11,1,13,3,4,5,6	Right carpus—slight medial displacement
G	January 18, 2019	Down	11,1,13,3,4,6	Right antebrachium—swelling. Possible muscle damage, infection, or bone remodeling
H^a^	April 27, 2019	Up	6,1,11	Right carpus—lateral displacement. Possible ligament damage
I^b^	August 31, 2019	Down	1,13,3,4,6	Left antebrachium—swelling. Possible muscle damage, infection, or bone remodeling
J	September 22, 2019	Down	11,12,1,13,3,9	Left tarsus—posterior swelling. Possible Achilles tendon damage
K^b^	April 30, 2020	Up	6,5,4,2	Left antebrachium—swelling. Possible muscle damage, infection, or bone remodeling
L^a^	May 01, 2020	Up	3,2,11	Right carpus—lateral displacement. Possible ligament damage

*Note*: Injury hypotheses were generated following consultation with vertebrate morphologist Hans Larsson, surgeon Ron Stradiotto, and veterinarian Mary Whitehall. Injury locations are quite clear, whereas the hypotheses amount to informed speculation. Videos are supplementary material video S1, and are also available at https://www.youtube.com/watch?v=ly1jVA6w5WM.

^a^ Possibly the same individual.

^b^ Possibly the same individual.

We generally expect that the injuries are musculoskeletal (as opposed to—for example—cutaneous), acquired (as opposed to congenital), and acute/traumatic (as opposed to chronic/gradual). However, without being able to examine internal anatomy of the injuries, it is important to outline the alternatives. First, certain diseases or parasites can cause “infectious lameness” evident as an altered gait even in the absence of musculoskeletal damage (Duncan & Angell, 2019) or by causing musculoskeletal damage (for an example in moose, see Honour & Hickling, 1993). Although lesions or external skin damage were not evident for the 12 injured moose, we cannot exclude the possibility of localized infections that compromise movement. Second, congenital diseases can cause angular limb deviations that sometimes persist into adulthood, in least in domestic animals such as cows (Abdelhakiem & Elrashidy, 2017) and horses (Witte & Hunt, 2009). We cannot rule out this possibility; yet the fact that we did not detect any injuries on yearling moose suggests that the injuries on adult moose were acquired rather than congenital. Third, repeated chronic stress can cause a gradual increase in locomotor impairment (Ely et al., 2009) and older moose often suffer from osteoarthritis (Peterson et al., 2010); yet we cannot assess the stage of healing or the age of the injured moose in our study. Although we consider these alternatives to be less likely than acquired acute/traumatic musculoskeletal damage, the ultimate cause of the injuries does not materially change our assessment of whether they compromise patterns of movement in wild moose.

Similar to uninjured moose, the injured moose moved downstream in the fall and upstream in the spring (Figure 5). The typical dates of movement of the injured moose also did not stand out from the entire distribution of moose observations (Figure 6). Although the two injured moose in fall 2019 moved earlier than did most other moose, the four injured moose in fall 2017 and 2018 tended to move rather late compared to the other moose. Of course, it is possible that these opposing (early or late) tendencies indicate that injured moose do deviate from the “typical” fall movement dates—just not in a consistent way. That is, injured moose might travel earlier *or* later than other moose—but this more detailed inference would require more data. By contrast, none of the injured moose appeared to move upstream in the spring on particularly early or late dates (Figure 6). On finer temporal scales, injured moose also did not stand out from the rest of the distribution: that is, some injured moose transited the trail on days when many other moose were transiting, some moved on days when modest numbers of other moose were transiting, and some moved on days when few other moose were transiting.

**FIGURE 7:**
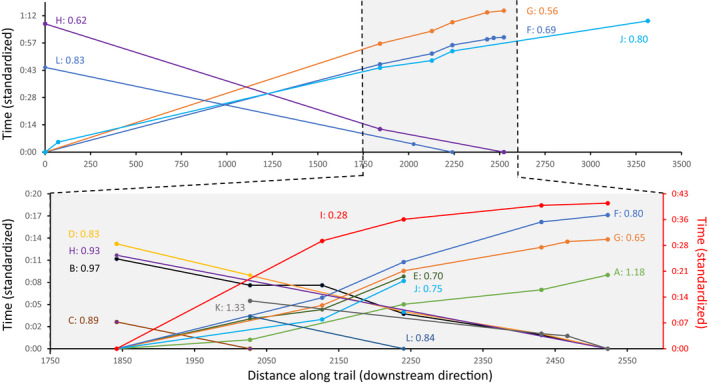
Cumulative time‐by‐location profiles for the 12 injured moose as indicated by capital letters that correspond to those shown in Figure 1. Time is standardized to zero at the first trap each moose was observed, with the traps ordered along the *x*‐axis according to their position along the trail. The cumulative time for each moose to pass each subsequent trap (on which it was observed) is then shown with the colored lines. Moose with lines rising from right to left were moving upstream on the trail, whereas moose with lines rising from left to right were moving downstream on the trail. The top panel shows the entire trail and the five moose that were observed at either the upstream end or the downstream end of the trail. The lower panel is an expanded version of the core center section of the trail where all 12 injured moose were observed. After the letters identifying each moose, we report the average rate of movement in m/s based on the two most distant traps each moose was observed in that panel. The scale in the lower panel is different for moose I—and is shown at right in red.

For the 12 injured moose, we calculated rates of travel along the trail by reference to the times they passed each camera trap (e.g., Rowcliffe et al., 2016). The resulting cumulative time‐by‐location profiles for these moose are shown in Figure 7, and the average rate of movement for each individual was calculated based on the two most widely separated traps on which that individual was recorded. These movement profiles and rates could be specified for all 12 injured moose across the core area of the study (traps 1–6 and 13: Figure 7), where estimates ranged from 0.28 to 1.33 m/s, with a mean and standard deviation of 0.85 ± 0.26 m/s. The obvious outlier was the injured moose that traveled at only 0.28 m/s. However, the time‐by‐location profile for this moose revealed that its slow pace was entirely due to an atypically long period between traps 1 and 13 (0.16 m/s), whereas its rate of movement between traps 13 and 6 was more typical (0.63 m/s).

Five of the 12 injured moose were also recorded at the upstream end of the trail (traps 11 and 12) and one of those moose was also recorded at the downstream end of the trail (trap 9). (Note: detectability appears lower at these upstream‐most and downstream‐most locations because moose sometimes there divert from the trail into the surrounding river flats.) Given that these upstream‐most and downstream‐most traps were >2 km apart, they presumably yield best estimates of the average rate of movement along the entire trail. The rate estimates for these five injured moose over this longer distance averaged 0.70 ± 0.11 m/s. (*Note*: the rates of movement for these five individuals over the shorter distances in the core area are also calculated and reported in Figure 7.) No obvious differences were evident in the rate of movement in the upstream versus downstream direction, although sample sizes are too small to warrant the estimation of mean rates of travel in each direction (Figure 7).

The injured moose clearly get along—but do they transit the trail at a slower rate than the uninjured moose? To answer this question, we would ideally generate rates of movement for many individual moose along the trail, and then compare the movement of injured moose to the corresponding distribution for uninjured moose. However, it is very difficult to confidently identify—across multiple camera traps—individual moose that do not have obvious identifying features, such as injuries. Furthermore, rates of movement could vary through the season, thus biasing comparisons to injured moose that traveled particularly early or late in the season. To circumvent these concerns, we compared individual injured moose with individual uninjured moose that passed the same camera traps within a few days of each other; although not at the same time so as to ensure they were moving independently (as opposed to traveling together). For 11 of the 12 injured moose, we were able to identify a "paired" uninjured moose passing the same traps within at least 4 days; and, for 3 of those 11 injured moose, we could identify two individual uninjured moose—one moving before and one moving after the injured moose. We then calculated the travel time between the two most widely spaced traps on which the injured and uninjured moose in each pair were recorded.

**FIGURE 8:**
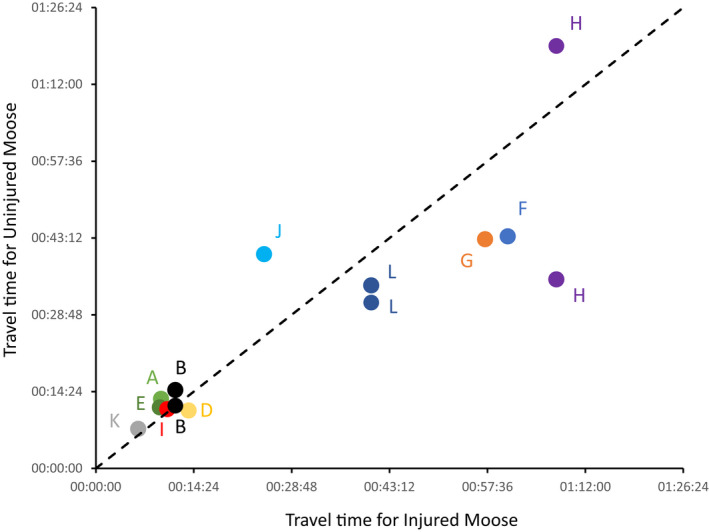
Comparison of rates of movement for 11 injured moose that could be paired with uninjured moose passing the same camera traps in the same direction within 4 days of each other. (The 12th injured moose could not be paired with an uninjured moose, and so is not shown here). For each injured‐uninjured pair of moose, the data show the transit time between the two most distant traps on which both moose of that pair were recorded. We were able to pair three of the injured moose (B, L, and H) with two uninjured moose each, one traveling before and the other after the injured moose ‐ both comparisons are shown for each of these "pairs." The dotted line is the 1:1 line of transit time for the injured and uninjured moose.

Over short distances (the core area of the set 1 traps 1–6), no obvious difference was evident in the rate of travel of injured and uninjured moose (Figure 8). Over longer distances that included the upstream‐most or downstream‐most traps, a trend toward somewhat slower movement for injured moose was suggested (Figure 8)—but any such trend was modest and remains uncertain given the low sample size.

As noted previously, most of our data do not meet the assumptions required for inferential statistics, and so the above interpretations are based on qualitative assessment of descriptive statistics and visual examination of data distributions—as seen in the figures. However, we appreciate that some readers might chafe at the absence of formal statistical support for our assertion that injured and uninjured moose do not differ in their movement. The one type of data that we feel can be formally analyzed is the relative rate of movement of injured and uninjured moose—that is, the 11 injured/uninjured pairs that are independent of each other, at least within a season. Hence, we analyzed the movement times shown in Figure 8 using only the first uninjured moose in cases where we could pair an injured moose with two uninjured moose. For each pair, we calculated the relative difference in time it took for the two moose to transit the two most distant traps on which they were both observed. We then compared these signed (negative when the injured moose was slower, positive when the injured moose was faster) proportional differences (difference in transit time between the two moose in a pair divided by the transit time of the slower moose in that pair) in a one‐sample t‐test (IBM SPSS Statistics 27) for whether the relative difference in speed was different from zero. This analysis statistically supports our assertion that rates of movement do not differ between injured and uninjured moose (*t*_10_ = 0.147, *p* = .886).

Overall, then, we suggest no detectable effect of these injuries on the transit of moose along the trail. Of course, we have not assessed other aspects of movement, such as whether injured or uninjured moose are more or less likely to use the trail that we instrumented with our camera traps. Furthermore, we cannot assess the movement of injured moose when they are not transiting between the upper and lower parts of the river, nor can we assess other components of their fitness, such as reproductive success or lifespan. Such comparisons would require other camera trap deployments and additional methodologies.

## 5 DISCUSSION

Major structural (bone, ligament, or tendon) injuries to moose limbs would seem incompatible with survival in the wild—and yet 1.4% of all observations of moose revealed such limb injuries. (We cannot be sure that all of the injuries were musculoskeletal—because we cannot examine internal anatomy of the injuries.) This injury percentage is much lower than those reported for structural limb injuries in other animals, such as 47.9% limb abnormalities in bonobos (Kano, 1984), 5.9% limb factures in seven small mammal species (Stephens et al., 2018), and 31% “moderate to severe” foot injuries in wolves (O'Brien et al., 2022). The frequency of leg injuries reported in some racehorse settings is similar (1.9%: Pieszka et al., 2011) to the one we observed in moose, although injuries appear higher in other racehorse settings (Ely et al., 2009). Also, Peterson et al. (2010) reports high frequencies of osteoarthritis in Isle Royale moose ‐ but it isn't clear if this degeneration would cause to angular limb displacements like those we documented.

The relatively low rate of injuries in moose presumably reflects the difficulty of post‐injury survival for such a heavy prey animal that sometimes needs to move quickly. Indeed, the observed frequency of injuries in moose (or other animals) is not a measure of the frequency at which such injuries occur. We would expect the latter frequency to be much higher because most moose suffering from them would die quickly due to predation or starvation—before our cameras could record them. On the other hand, some moose with such injuries might recover so well that their injuries are no longer detectable by the time our cameras recorded them. Regardless, the frequency of seemingly severe injuries on ambulatory moose in nature was surprising—at least to us. The location and nature of these injuries was variable (Figure 1) and their specific cause (e.g., damaged ligaments or tendons, bone remodeling, infection, arthritis) could only by hypothesized (Table 1).

Our identification of injured moose was based on obvious physical signatures (e.g., swelling or displacement) rather than on how they were moving. And yet those injuries did influence immediate movement behavior because every injured moose was limping and one (moose H) showed—in all observations—the expected behavioral signs of pain, such as flattened ears and extended nostrils (Costa et al., 2014). Surprisingly, however, the injured moose did not stand out from the uninjured moose in any of the variables we measured, including the direction (Figure 5), date (Figure 6), contagiousness (Figure 6), and speed (Figure 8) of movement. Furthermore, some of the 12 injured moose were probably the same individual recorded in different years or seasons (Table 1), suggesting survival over extended periods of time. For example, based on detailed examining of the videos, we suggest that moose B (2018)—and especially moose H (2019) and L (2020)—could all be the same moose. Further, several of the injured moose (A, G, and K) were trotting in some of the videos—as we also sometimes see for uninjured moose. Finally, it was interesting to note that the only recorded instance of aggression between moose was of an injured moose (B) using its injured leg to kick at an uninjured moose (visible on the videos embeded here and on YouTube).

Some animals with structural leg injuries are known to survive for extended periods in the wild; yet these instances seem most common for small animals, for young animals cared for by their parents, or for social animals that receive group protection and benefits. For instance, more than 5% of small mammals show evidence of healed bones (Stephens et al., 2018) and three‐limbed primates and canids have been recorded living for extended periods in nature (O'Brien et al., 2022; Waller & Reynolds, 2001). (As an aside, we frequently see multiple individuals in wolf packs limping in our videos.) The situation would be very different for the adult moose we recorded because they are large and not in social support groups that can provide a “soft landing.” Although some of the injured moose we observed might have been injured and then recovered when still with their mothers, we did not document any obvious injuries on moose calves.

How can we explain the apparent success of these moose with what appear to be serious limb injuries? One hypothetical explanation might be a lack of predators (e.g., Palombo & Zedda, 2016); and yet wolves, including in packs of more than 10 individuals, were the second most frequent observations on our camera traps (Figure 4). Wolves are known to be important predators on moose in northern British Columbia (e.g., Larsen et al., 1989)—although predation rate estimates are not available for the Kispiox River watershed. Instead, the most straightforward explanation might be that moose with particularly debilitating injuries died before we recorded them. That is, only individuals whose injuries did not dramatically compromise locomotion—at least not for a long period of time—were able to survive and thus be recorded by our cameras. Although we cannot be certain of the precise nature of the injuries we recorded, we do suggest they reflect damage to ligaments rather than bones—although subsequent bone remodeling seems likely. It is also possible that some moose were simply “lucky” immediately after the injury to not have been found by wolves. Interestingly, we saw similar numbers of injured moose moving upstream and downstream, indicating that survival was not dependent on being present in either the upstream or downstream areas. Of course, it is important to note that our observation of ambulatory moose with leg injuries does not equate to their fitness—that is, “performance” of these moose still might be impacted when it comes to mating or calf rearing or long‐term survival.

We have written an entire paper about, in essence, only 12 individuals—some of which could be the same individual recorded in different years/seasons. Although this sample size is the largest analyzed for live injured moose in a wild population (unless you count osteoarthritis documented after death: see Peterson et al., 2010), we have taken care to caution against extrapolation to other populations or species. Fortunately, the use of camera traps is becoming very common for monitoring wild animal populations, and so it is possible that data and analyses similar to ours could be attempted in other camera trap studies. In order to facilitate such work, we recommend that more monitoring studies record video observations (see also Caravaggi et al., 2017), rather than just photographs—the latter currently being standard for population assessment (Palencia et al., 2021) but obviously limiting when it comes to quantifying some aspects of animal behavior.

## 6 BROADER CONSIDERATIONS

How might injuries influence natural selection and evolution? First, some contributors to injury are not stochastic but rather are shaped by genetically based traits and behaviors, which should then evolve to reduce the chances of a debilitating injury. This form of selection is presumably why—for example—bones, teeth, and beaks are so hard and resistant to fracture (Christiansen & Adolfssen, 2005; Soons et al., 2015). Hence, we must assume that past selection to avoid injury has shaped the leg morphology and behavior of moose such that those injuries are not common. Indeed, we have recorded many instances of moose tripping on logs or sticks (especially at night or when running) or slipping on ice; and yet, in each case, they quickly recovered their balance without apparent harm. Of course, natural selection will not completely eliminate the possibility of injury for three reasons. First, trade‐offs presumably exist with injury prevention, such as the need to forage in risky situations and the extra weight associated with structures that are over‐built. Second, once injuries become relatively rare, selection for further injury prevention would weaken, and so the evolution of unbreakable structures would not be expected. Third, some injuries might be so stochastic in origin.

Stochastic injuries amounting to “bad luck” are not expected to be associated with genetic variation in organismal traits. Hence, such injuries might be expected to weaken natural selection—as will other sources of randomness in mortality or mating success (Haller & Hendry, 2014; Snyder & Ellner, 2018). We suggest that this effect of injuries on natural selection might be quite substantial. In our case, more than 1% of all observations of moose revealed obvious injuries, suggesting that the true frequency of such injuries must be much higher—and it is indeed high in some other wild animals (e.g., Kano, 1984; O'Brien et al., 2022; Stephens et al., 2018). Thus, stochastic injuries might be a major source of randomness in evolution that ultimately limits the power and precision of natural selection. We suggest the value of additional work on the frequency of injuries, the extent of recovery from those injuries, and the overall effects on natural selection—both sharpening (selection to avoid injuries) and dulling (due to stochastic injuries).

## AUTHOR CONTRIBUTIONS

**Andrew Hendry:** Conceptualization (equal); data curation (lead); formal analysis (lead); funding acquisition (lead); investigation (equal); methodology (equal); project administration (lead); resources (lead); supervision (lead); visualization (lead); writing – original draft (lead); writing – review and editing (lead). **Aspen Sierra Hendry:** Conceptualization (equal); data curation (supporting); investigation (supporting); methodology (equal); writing – original draft (supporting); writing – review and editing (supporting). **Heather Lynn Roffey:** Conceptualization (equal); data curation (supporting); investigation (equal); methodology (equal); writing – original draft (supporting); writing – review and editing (supporting). **Cedar Alyce Hendry:** Conceptualization (equal); data curation (supporting); investigation (supporting); methodology (equal); writing – original draft (supporting); writing – review and editing (supporting). **Michael Hendry:** Conceptualization (equal); methodology (equal); resources (supporting); writing – original draft (supporting); writing – review and editing (supporting).

## CONFLICT OF INTEREST

The authors are all members of the same family—but did not receive any financial support from research grants, government agencies, NGOs, or businesses.

## Supporting information


Video S1

## Data Availability

The data on which the analyses were conducted have been deposited in DRYAD: https://doi.org/10.5061/dryad.xd2547dkk.
